# Time-lapse imaging reveals highly dynamic structural maturation of postnatally born dentate granule cells in organotypic entorhino-hippocampal slice cultures

**DOI:** 10.1038/srep43724

**Published:** 2017-03-03

**Authors:** Tijana Radic, Tassilo Jungenitz, Mathias Singer, Marcel Beining, Hermann Cuntz, Andreas Vlachos, Thomas Deller, Stephan W. Schwarzacher

**Affiliations:** 1 Institute of Clinical Neuroanatomy, NeuroScience Center, Goethe University, Frankfurt am Main, Germany; 2 Ernst Strüngmann Institute (ESI) for Neuroscience in Cooperation with Max Planck Society, Frankfurt am Main, Germany; 3 Frankfurt Institute for Advanced Studies (FIAS), Frankfurt am Main, Germany

## Abstract

Neurogenesis of hippocampal granule cells (GCs) persists throughout mammalian life and is important for learning and memory. How newborn GCs differentiate and mature into an existing circuit during this time period is not yet fully understood. We established a method to visualize postnatally generated GCs in organotypic entorhino-hippocampal slice cultures (OTCs) using retroviral (RV) GFP-labeling and performed time-lapse imaging to study their morphological development *in vitro*. Using anterograde tracing we could, furthermore, demonstrate that the postnatally generated GCs in OTCs, similar to adult born GCs, grow into an existing entorhino-dentate circuitry. RV-labeled GCs were identified and individual cells were followed for up to four weeks post injection. Postnatally born GCs exhibited highly dynamic structural changes, including dendritic growth spurts but also retraction of dendrites and phases of dendritic stabilization. In contrast, older, presumably prenatally born GCs labeled with an adeno-associated virus (AAV), were far less dynamic. We propose that the high degree of structural flexibility seen in our preparations is necessary for the integration of newborn granule cells into an already existing neuronal circuit of the dentate gyrus in which they have to compete for entorhinal input with cells generated and integrated earlier.

In the dentate gyrus (DG) of the hippocampus, neurogenesis is maintained throughout the lifetime of mammals, including humans^1^,^2^. New neurons generated from neural stem cells (NSCs) in the subgranular zone (SGZ) are structurally integrated into the network of mature granule cells (GCs) and ultimately play an important role in hippocampus-dependent functions related to learning and memory as well as mood regulation^2^,^3^,^4^,^5^,^6^,^7^,^8^. Disturbances in this process may lead to various pathological manifestations including serious disorders such as dementia, epilepsy, and depression^9^,^10^,^11^,^12^,^13^. Due to the tremendous potential of NSCs in regenerative therapy, it is crucial to examine the fundamental processes which regulate proper maturation and integration of newborn GCs into the pre-existing hippocampal circuitry.

The development of the DG in rats is generated in three distinct phases^14^. First NSCs derive from the primary dentate neuroepithelium and proliferate near the lateral ventricle around embryonic day 16 (E 16). By E 18, the secondary dentate matrix emerges in the subventricular area which consists of proliferative cells that migrate toward the formative DG in the so-called “first dentate migration” and initially form the outer parts of the suprapyramidal blade of the granule cell layer (GCL), then add GCs to the infrapyramidal blade. By E 22, a different pool of migratory precursors, the “second dentate migration,” begins to contribute GCs to the inner layers of the GCL. In the third phase, the tertiary dentate matrix arises at postnatal day 1 (P 1) in the dentate hilar region and consists of cells that divide and settle locally within the area that is to become the SGZ^14^,^15^. Proliferation of postnatal GCs in this tertiary matrix reaches its peak at P 5–8^16^ and continues to take place throughout life^14^,^17^. By P 5, both blades of the DG have largely been established, though the infrapyramidal blade is still expanding and consists of more immature GCs than the suprapyramidal blade. New GCs are continuously added to the inner GCL layers by both the second dentate migration and the tertiary matrix that is now prominent in the formative SGZ^14^. Recently, it has been suggested that the adult neurogenic niche in the SGZ emerges as a continuation of peri- and postnatal development of the DG between P 7 and P 14, much earlier than adulthood is reached by the animals^17^. Although the neurogenesis process seems to be progressing at a slower pace in the adult hippocampus, it shows many similarities to the embryonic development of GCs^7^,^18^,^19^. While the time course of development is variable between individual GCs^20^,^21^,^22^, in general, newly generated GCs in the postnatal brain need at least 5 weeks to mature and become fully integrated into the existing network^18^,^19^. Current knowledge on the morphological development of newborn GCs is predominantly based on data obtained after the fixation of brains at different time points^18^,^19^,^20^,^22^,^23^. In order to examine detailed cellular dynamics and regulatory processes during the course of structural cell development within the hippocampal neurogenic niche, however, a system that allows the imaging of individual cells over time is needed.

In this study we have tested the possibility to study neurogenesis in organotypic slice cultures *in vitro*. So far, only few such investigations have been performed in this or in other *in vitro* systems and their usefulness has been controversially discussed^24^,^25^,^26^,^27^,^28^. Here, we established an *in vitro* method employing the organotypic entorhino-hippocampal slice culture (OTC) technique^29^ combined with retroviral (RV) and adeno-associated viral (AAV) labeling in order to visualize and follow individual postnatally born, as well as older GCs, over different periods of development using time-lapse imaging. Three-dimensional computer reconstructions of complete imaged GCs revealed a remarkably dynamic course of dendritic development in newborn GCs, including elongation as well as retraction of processes. In contrast, more mature GCs exhibited a relatively stable dendritic arbor with only little change over time. We hypothesize that this dynamic morphological remodeling is needed for the successful structural and functional integration of newborn GCs into an existing cellular network. Furthermore, we propose that the analysis of postnatal neurogenesis in OTCs will make it possible to identify mechanisms of structural development and functional integration by analyzing changes of single identified newborn GCs over time.

## Results

### Immunocytochemical characterization of postnatal neurogenesis in OTCs

OTCs were prepared at P 4–5 using the interface method^30^. Brains were sliced in the horizontal plane with a vibratome into 300 μm thick sections. Subsequently, the hippocampi with attached entorhinal cortices were dissected, placed onto semiporous membrane culture inserts, and cultivated in serum-containing medium. To assess the histology of postnatal OTCs, we explored the expression of the immunocytochemical markers DCX for immature neurons and calbindin for mature neurons. To specifically identify GCs, we used the GC marker Prox1.

**Figure 1:**
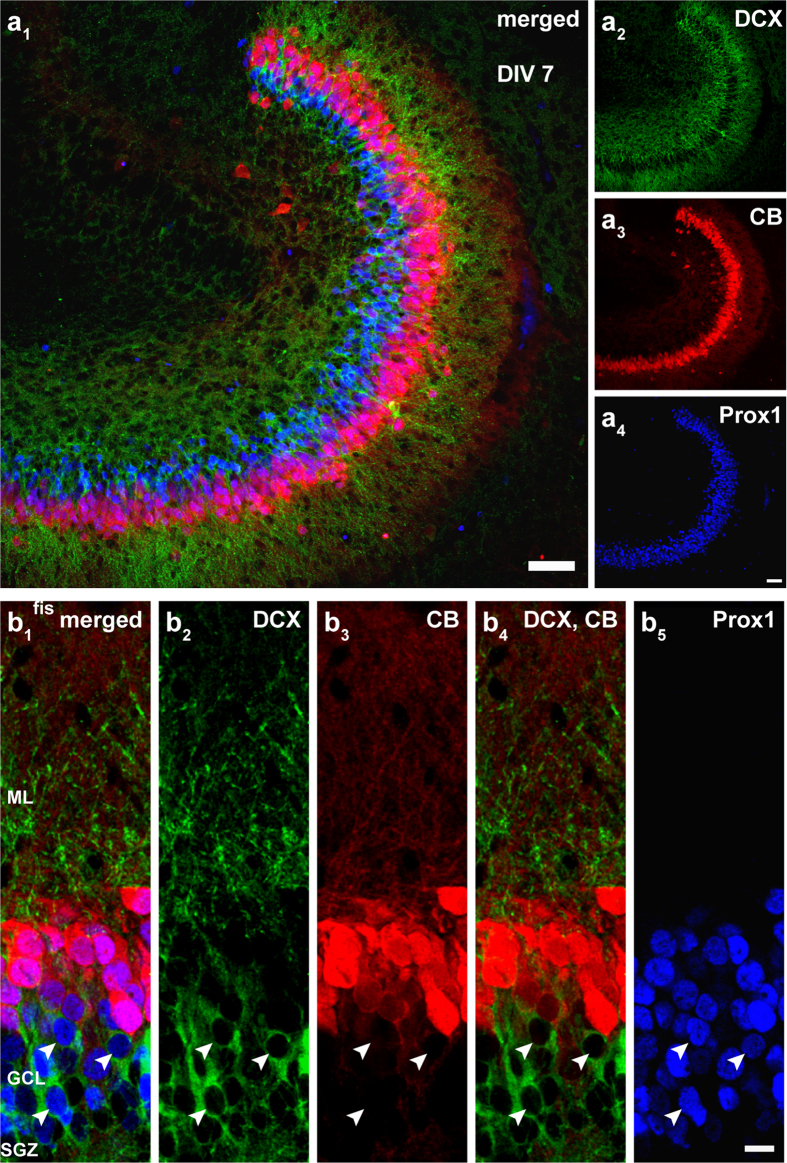
Immunocytochemical evaluation of postnatal OTCs at DIV 7. (**a**) Resliced OTC sections (50 μm) prepared on P 4-5 and fixed on DIV 7 were processed for immunoreactivity of the immature neuronal marker doublecortin (DCX; green), the mature neuronal marker calbindin (CB; red), and the granule cell marker Prox1 (blue). (**b**) Expression of DCX was observed in the subgranular zone (SGZ), the inner and middle parts of the granule cell layer (GCL) in cell somata, as well as dendrites that extended into the molecular layer (ML). Calbindin labeling was detected in dentate granule cells (GCs) that were located in the outer parts of the GCL with processes that reached the hippocampal fissure (fis). Prox1 was expressed in both, immature and more mature GCs in the GCL. DCX and calbindin co-localized only rarely, while Prox1 was co-expressed with both of these markers in addition to being present in other GCs as well. Scale bars: (**a**) 50 μm; (**b**) 10 μm.

At day *in vitro* (DIV) 7, we detected widespread DCX expression throughout the SGZ and most of the granule cell layer (GCL), while some DCX-expressing cells were also found in the hilus (Fig. 1a). Calbindin immunoreactivity was observed solely in cells that were located in the outer part of the GCL (Fig. 1). DCX and calbindin rarely co-localized which indicates that DCX marked young, immature neurons while calbindin was only expressed in more mature GCs. This finding reflects the typical layering of immature neurons being located in the SGZ and the inner part of the GCL, while mature neurons are found in the outer part of the GCL, toward the molecular layer (ML)^22^,^31^. The fact that at DIV 7 we found more DCX-positive cells relative to calbindin-positive cells is also congruent with the fact that this is still the time period of high cell proliferation and neurogenesis^16^. In addition, we examined the distribution of Prox1 which is typically present in granule cells from an early time point on^32^ and found that it was indeed expressed in cells throughout the GCL, including all DCX and calbindin-labeled neurons (Fig. 1b). These findings reveal that Prox1 is a useful marker of both immature and mature GCs in OTCs at DIV 7.

**Figure 2:**
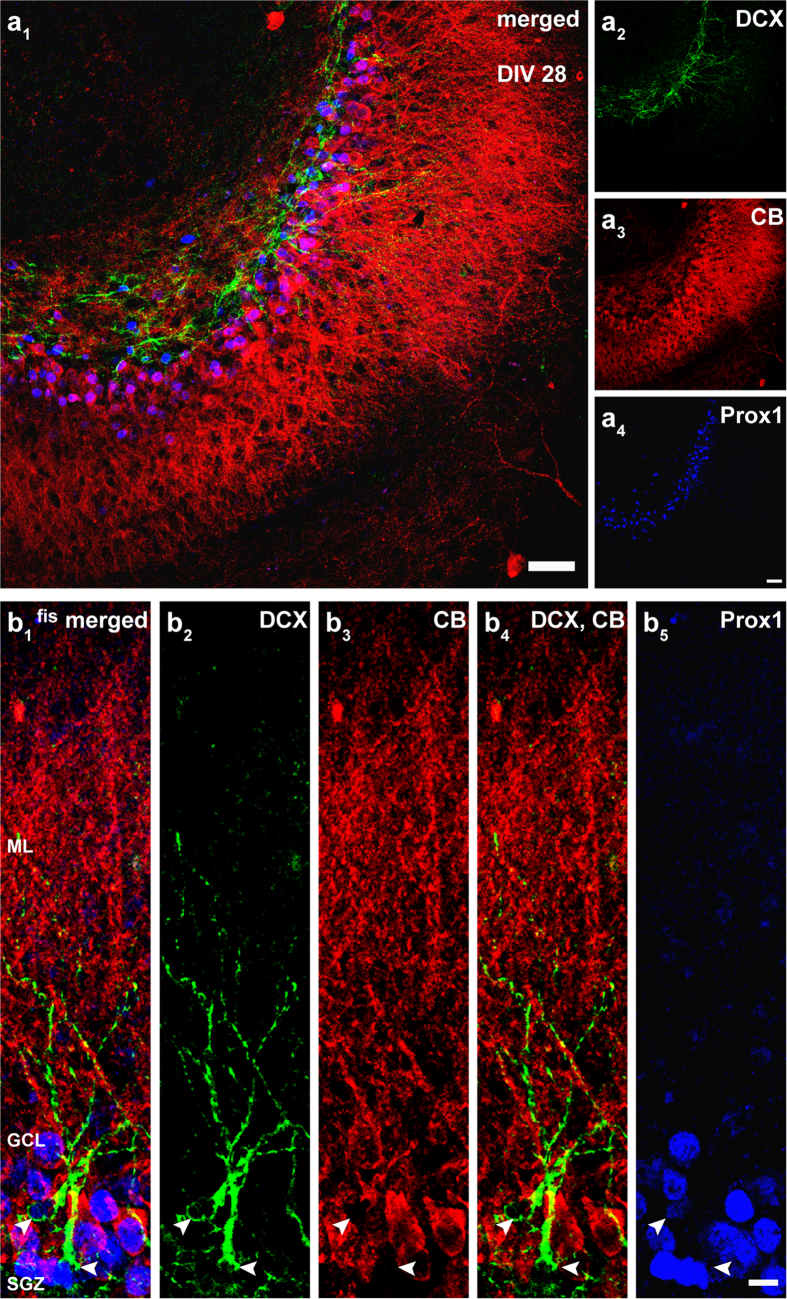
Immunocytochemical evaluation of postnatal OTCs at DIV 28. (**a**) Immunostainings of OTC sections fixed on DIV 28 displayed extensive expression of calbindin (red) throughout the granule cell layer (GCL), while there were only few DCX-positive (green) cells in the subgranular zone (SGZ). Prox1 (blue) was expressed in the entire GCL in immature and mature GCs. (**b**) Few DCX-positive cells were found in the SGZ in 28-day-old OTCs. Most GCs were calbindin-positive and extended their dendrites through the molecular layer (ML) to the hippocampal fissure (fis), indicating that at this time point, most GCs are mature and the neurogenesis rate has decreased. Scale bars: (**a**) 50 μm; (**b**) 10 μm.

We applied the same markers to OTCs cultured until DIV 28 and found that at this time point, the number of calbindin-positive cells increased in the GCL, while in the inner part a smaller number of cells expressed DCX (Fig. 2). Prox1 was again present in both, DCX- and calbindin-positive GCs. The low number of DCX-positive GCs indicated that the rate of neurogenesis had decreased over the 3-week period. However, the fact that we observed DCX-positive cells at DIV 28 in OTCs also implied that new cells continued to be generated in culture after several weeks *in vitro*, albeit in lower numbers.

Overall, our results suggested that OTCs displayed characteristic expression patterns of maturation markers, hence indicating that OTCs exhibited an organotypic organization at different postnatal time points.

### Retroviral transduction of newly born dentate GCs in OTCs by local injection

A retroviral (RV) vector based on the Moloney murine leukemia virus (MMLV) containing the gene for GFP under the CAG promoter^19^,^33^ was used to label newborn cells in OTCs. The RV solution was injected directly into the hippocampus at DIV 0 (OTC preparation at P 4–5). Prox1 and DCX were used after fixation to identify RV-transduced GCs.

**Figure 3:**
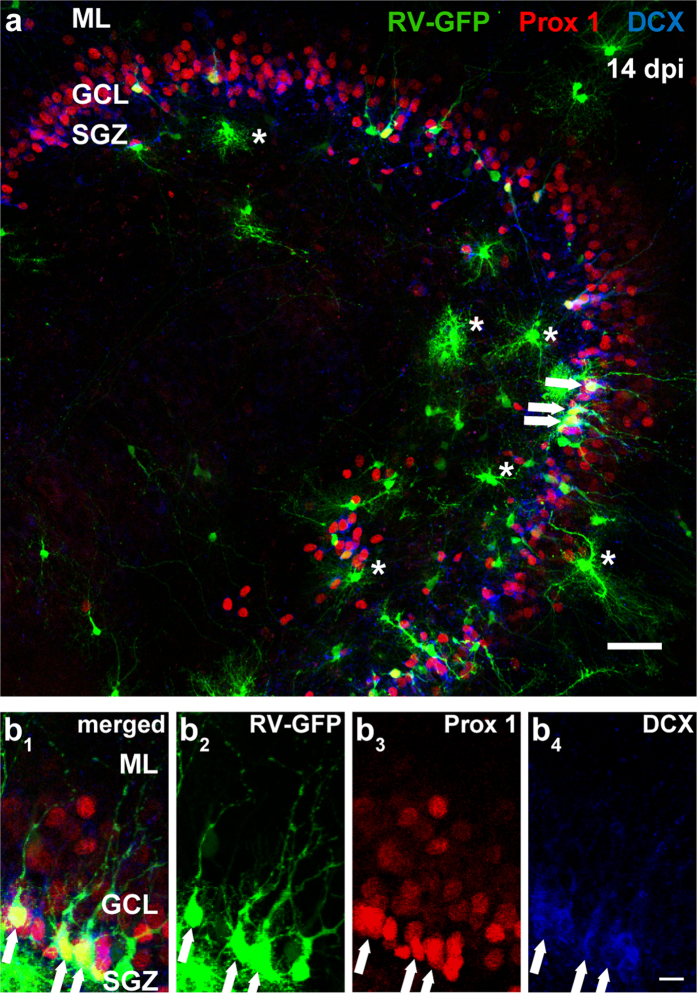
Retroviral (RV) transduction of newborn dentate granule cells (GCs) by local injection. (**a**) A triple immunostaining of an OTC that was transduced with RV-GFP on DIV 0 and fixed on 14 dpi. DCX immunoreactivity was observed in the subgranular zone (SGZ) and the inner part of the granule cell layer (GCL), while Prox1 was expressed throughout the GCL. Several RV-GFP-labeled cells located in the SGZ and inner GCL were polarized with processes extending into the GCL and the molecular layer (ML), and co-expressed Prox1 and DCX (white arrows), suggesting that a number of newborn GCs were transduced with the RV. In addition, there were RV-GFP-positive cells with glial morphology that did not express DCX, nor Prox1, (examples are denoted by asterisks). (**b**) A magnification shows RV-GFP-labeled cells located in the SGZ and the inner layers of the GCL co-expressing both Prox1 and DCX (white arrows). Scale bars: (**a**) 50 μm; (**b**) 10 μm.

Figure 3a illustrates an example of an RV-transduced OTC that was fixed at 14 days post RV injection (14 dpi). Prox1 expression was detected throughout the entire GCL and occasionally in the hilus. A number of RV-GFP-positive cells displayed co-localization of Prox1 and DCX, showing successful RV-transduction of maturing dentate GCs (Fig. 3). At the cell age of 14 days, RV-labeled GCs displayed a soma located in the SGZ or the inner part of GCL, and several polarized neurites oriented toward the GCL and reaching into the ML. The labeled processes did not contain any obvious spine-like structures at this time point. In addition to RV-labeled GCs, we observed a number of RV-GFP-positive cells with glial morphology that did not express DCX, nor Prox1 (marked with asterisks in Fig. 3a) indicating that due to the cell-unspecific promoter CAG, the RV-GFP was incorporated in newborn neurons as well as glial cells. Based on specific morphological features and the absence of neuronal markers, glial cells could easily be distinguished from RV-labeled neurons.

### Anterograde tracing shows presence of entorhinal projection fibers in the molecular layer of OTCs during the development of newborn GCs

**Figure 4:**
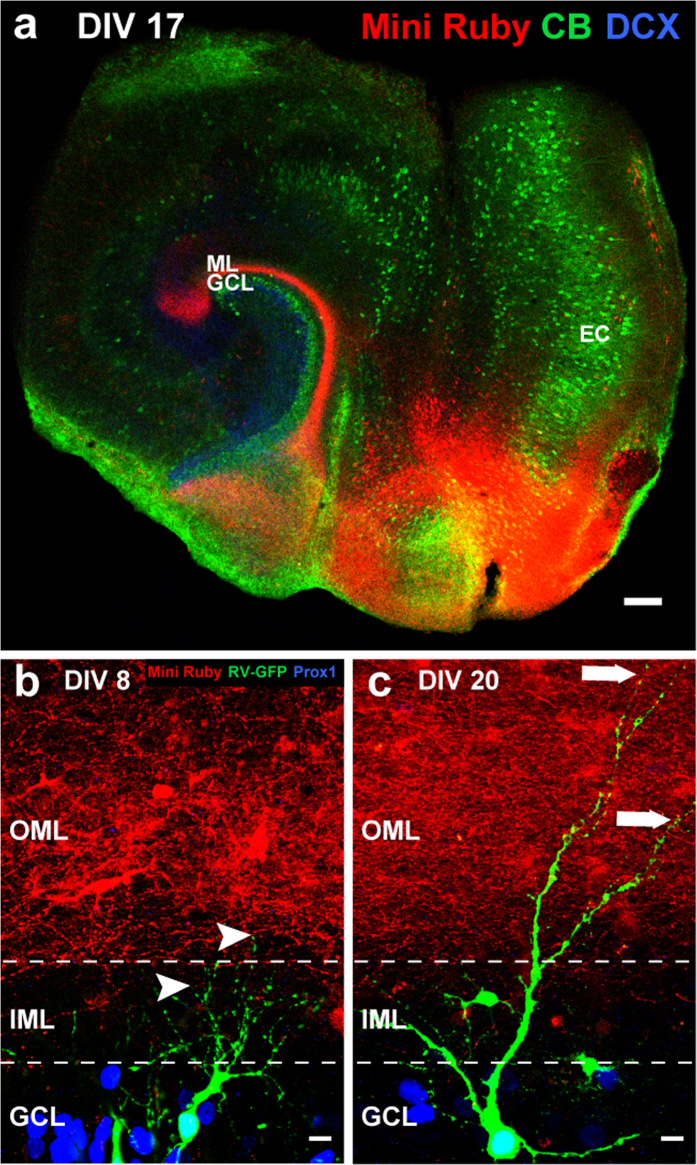
Perforant path fibers are present in the outer molecular layer (OML) during the development of RV-GFP-labeled GCs *in vitro*. (**a**) The biotinylated dextran amine tracer Mini Ruby was applied to the entorhinal cortices (ECs) of OTCs at DIV 15 and images were taken on DIV 17 showing the axons of EC neurons projecting to the molecular layer (ML) of the dentate gyrus (red). In the granule cell layer (GCL), mature neurons are labeled with the marker calbindin (CB), while immature neurons are labeled with doublecortin (DCX). (**b**) Z-projection of two images showing an 8-day old OTC treated with the RV (green) and Mini Ruby (red). Mini Ruby was applied on DIV 3 and OTCs were fixed on DIV 8. Prox1-positive (blue) 8-day-old postnatally born RV-GFP-labeled GCs directed their growing dendrites toward the OML (white arrowheads) that was already re-innervated by Mini Ruby-labeled entorhinal fibers. (**c**) Z-projection of four images (z-axis interval: 1 μm) of an RV and Mini Ruby treated OTC at DIV 20. A 20-days-old RV-GFP-labeled GC exhibiting an elaborate dendritic arbor that extended well into the OML (white arrows) where labeled perforant path axons were present. Scale bars: (**a**) 50 μm, (**b,c**) 10 μm.

The entorhinal cortex (EC) is a major source of synaptic input onto dentate GCs. Axons of EC neurons project through the perforant path to the hippocampus, including the molecular layer of the DG^34^,^35^. It has been shown that in OTCs, entorhinal axons are present in the DG shortly after explantation^36^,^37^. To study whether RV-labeled newborn GCs grow their dendrites into a pre-existing area of entorhinal axons - as is the case in adult neurogenesis - anterograde tracing was performed by placing the biotinylated dextran amine Mini Ruby on the surface of the EC on DIV 3 and DIV 15. Cultures were fixed at different time points between DIV 8 and DIV 20. Figure 4a shows an overview of Mini Ruby labeling in the EC neurons and their projections to the ML. Neuronal markers DCX and calbindin were used to visualize the immature and mature GC populations of the GCL, respectively. At early time points such as DIV 8, we observed Mini Ruby labeling of perforant path fibers in the OML of OTCs. Young, Prox1-positive 8-day-old GCs extended dendritic processes into the IML but did not reach the area of labeled entorhinal fibers in the OML yet (Fig. 4b). In contrast, at DIV 20, newborn GCs exhibited dendritic trees that extended into the OML and individual segments of these cells intermingled with Mini Ruby-labeled afferent fibers (Fig. 4c). We conclude that RV-labeled newborn GCs extend their dendrites into a field of entorhinal axonal terminations *in vitro*, as is also the case during adult neurogenesis.

### Time-lapse imaging of RV-labeled GCs

**Figure 5:**
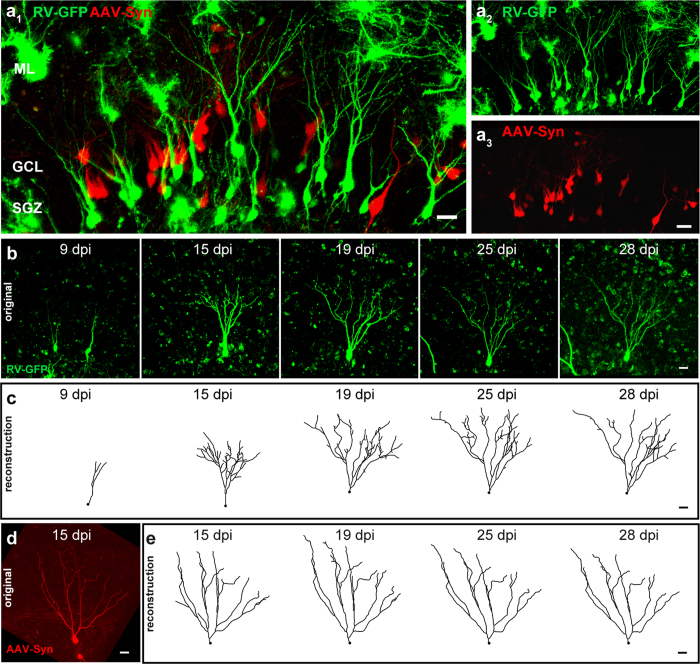
Time-lapse imaging and reconstruction of RV-CAG-GFP-labeled postnatally born GCs and older AAV-Synapsin1-tdTomato-labeled GCs. (**a**) OTCs were transduced with an RV-CAG-GFP and an AAV-Synapsin1-tdTomato vector to label newborn (RV-GFP; green) and older GCs (AAV-Syn; red) respectively. (**b,c**) Daily time-lapse imaging of individual RV-GFP-labeled GCs was performed over a period of three weeks between 8 and 28 days post virus injection (dpi). All cells were 3-D computer reconstructed to allow for detailed morphological analysis over time. (**d,e**) Example of an AAV-Syn-labeled cell with 3-D reconstructions at different time points. Scale bars: (a_1_) 20 μm, (a_2_-e) 10 μm.

Successful RV-labeling of postnatally born GCs in OTCs enabled live imaging of individual cells over extended periods of time. To obtain a perspective of the degree of dynamic restructuring of newborn GCs, we compared the morphological development of newborn RV-GFP labeled GCs with older, more mature GCs during the same time window and in the same preparation. To label older GCs, we used an adeno-associated viral (AAV) vector expressing tdTomato under the synapsin 1 promoter. Synapsin 1 is a protein that is involved in the clustering of synaptic vesicles and neurotransmitter release^38^,^39^,^40^ and is thus only expressed in neurons that have established synapses. OTCs were injected at DIV 0 (=postnatal day 4–5). Figure 5a displays the GCL of an OTC that was transduced with both, the RV-CAG-GFP (RV-GFP) and the AAV-synapsin1-tdTomato (AAV-Syn) vectors. While RV-GFP was expressed in newborn cells in the inner part of the GCL, tdTomato was detected in GCs that were not RV-GFP-positive, and located throughout the GCL.

Time-lapse imaging was performed from 8 to 28 dpi on at least 7 consecutive days in order to follow the dendritic branches of newborn GCs (n = 18 GCs from 16 cultures of 13 animals, mean time of observation 13.67 ± 4.77 days) during different stages of development (Fig. 5b). Sequential daily live observation of individual newborn GCs enabled analysis of changes in morphology within each cell using 3-D computer reconstruction (Fig. 5c) and thus detection of dynamic processes underlying structural GC development. Presumably, AAV-Syn transduced neurons were prenatally born and already more mature at the time of transduction. This assumption was supported by the fact that all tdTomato-positive cells exhibited a mature dendritic structure throughout the imaging period (Fig. 5d,e), even at early time points such as 8 dpi.

**Figure 6:**
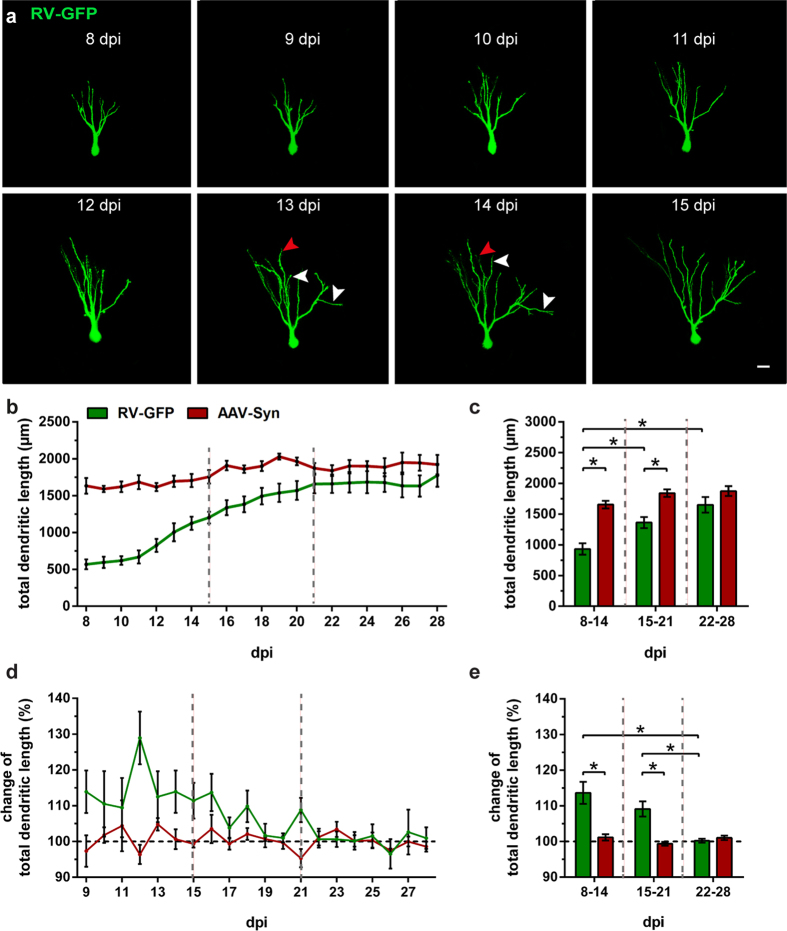
Newborn RV-GFP-labeled GCs exhibit higher dendritic restructuring and dynamics compared with older AAV-Syn-labeled GCs. (**a**) Time-lapse image sequence of a newborn cell on 8 consecutive days from 8–15 dpi. During this time frame, we observed a phase of high dynamics in structural rearrangement. While during the second week of development the cell exhibited considerable dendritic growth (white arrowheads), we also observed withdrawal of dendritic segments (red arrowheads), or complete branches. (**b**) Daily mean values of the total dendritic length (TDL) for RV-GFP-labeled GCs (green) and AAV-Syn-labeled GCs (red). (**c**) Mean TDL values grouped by week showed significant differences in RV-GFP-labeled GCs over time, i.e. between the second and the third week (two-way ANOVA with Bonferroni correction, P = 0.0016) as well as between the second and the fourth week (P < 0.0001). Moreover, the two cell populations displayed significant differences in the TDL during 8-14 dpi (two-way ANOVA with Bonferroni correction P = 0.0012) and 15–21 dpi (P = 0.0027). (**d**) To analyze dynamic changes in dendritic development, we calculated the average change in dendritic length per day and expressed the values as the percentage of the TDL of each previous day which was defined as 100%. Newborn GCs exhibited significant differences in TDL change between the first and the fourth week (two-way ANOVA with Bonferroni correction P < 0.0001) and between the third and the fourth week (P = 0.0053). (**e**) Newborn GCs displayed a significantly higher degree of change in TDL during 8–14 dpi (P = 0.0106) and 15–21 dpi (P = 0.0029) compared with older GCs. (**c,e**) 8–14 dpi: n_RV-GFP_ = 17 (**c**), 12 (**e**), n_AAV-Syn_ = 4; 15–21 dpi: n_RV-GFP_ = 18, n_AAV-Syn_ = 10; 22–28 dpi: n_RV-GFP_ = 11, n_AAV-Syn_ = 5. n represents number of cells (1–2 cells per culture). Error bars represent SEM. *P < 0.05. Scale bar: 10 μm.

Between 8 and 14 dpi, we observed a phase of considerable growth of neurites with a large expansion of the dendritic tree and branching (Fig. 6a). Starting at 11 dpi, the cells exhibited elaborated apical dendrites that ran through the GCL and into the ML where they divided into several orders of branches (Fig. 6a). Some cells exhibited basal dendrites that projected to the hilus. In most cases, we also observed axonal processes that extended toward the hilus where they showed branching of collateral fibers. Individual cells displayed both, extension of dendrites toward the ML (Fig. 6a, white arrowheads), as well as retraction of branches (Fig. 6a, red arrowheads). For a comparison of dendritic dynamics in older and newborn GCs, we performed time-lapse imaging and 3-D reconstructions of RV-GFP-labeled and AAV-Syn-labeled cells at 24 h intervals. AAV-Syn-positive GCs revealed a stable dendritic shape and structure over an extended period of time. The total dendritic length (TDL) remained unchanged throughout all time points (Fig. 6b,c). RV-GFP-labeled newborn GCs on the other hand displayed a clear increase of TDL between 8 and 21 dpi after which it remained relatively constant (Fig. 6b,c). Mean TDL values grouped by week showed significant differences in RV-GFP-labeled GCs over time, i.e. between the second and the third week (8–14 dpi: 931.33 ± 90.16 μm; 15–22 dpi: 1,361.94 ± 87.03 μm; two-way ANOVA: time effect F_(2,59)_ = 6.365, P = 0.0031, followed by *post hoc* Bonferroni’s test, P = 0.0016) as well as between the second and the fourth week (8–14 dpi: 931.33 ± 90.16 μm; 22–28 dpi: 1649.80 ± 121.03 μm; P < 0.0001). RV-GFP-labeled GCs exhibited a significantly lower TDL compared to AAV-Syn-labeled GCs between 8 and 14 dpi (RV-GFP: 931.33 ± 90.16 μm; AAV-Syn: 1,653.72 ± 54.29 μm; two-way ANOVA: group effect: F(1, 59) = 22.40, P < 0.0001, followed by *post hoc* Bonferroni’s test, P = 0.0012) and between 15 and 21 dpi (RV-GFP: 1,361.94 ± 87.03 μm; AAV-Syn: 1,840.86 ± 58.53 μm, P = 0.0027), whereas between 22 and 28 dpi, there was no significant difference in TDL between the two cell populations (Fig. 6c). These findings show that AAV-Syn-labeled cells exhibited a higher TDL early on and overall more structural stability compared with RV-GFP-labeled newborn GCs, which was presumably due to their advanced cell age and stage of maturation.

Because of the fact that the initial increase and subsequent plateau in dendritic length and complexity in RV-GFP-labeled newborn GCs was the result of a very dynamic process, we examined the dynamics of extension and retraction of dendrites in newborn GCs by calculating the average change in TDL per day during different time periods (Fig. 6d,e). These values were expressed as the percentage of the TDL value of each previous day which was defined as 100%. When values were grouped by week, we found a high amount of change in TDL during the second and third week in newborn GCs, whereas in the fourth week, there was almost no change. Significant differences occurred between the second and the fourth week (8–14 dpi: 113.64 ± 2.97%; 22–28 dpi: 100.21 ± 0.52%; two-way ANOVA: time effect: F_(2,54)_ = 2.963, P = 0.0601, followed by *post hoc* Bonferroni’s test, P < 0.0001) and between the third and the fourth week (8–14 dpi: 109.13 ± 2.05%; 22–28 dpi: 100.21 ± 0.52%, P = 0.0053; Fig. 6d,e). In contrast, AAV-Syn-labeled GCs did not display major changes in the TDL throughout the entire time period (Fig. 6d,e). As a result, RV-GFP-labeled GCs exhibited significantly higher changes in dendritic length between 8 and 14 dpi compared with older GCs (RV-GFP: 113.64 ± 2.97%; AAV-Syn: 101.15 ± 0.77%, two-way ANOVA: group effect: F_(1, 54)_ = 11.73, P = 0.0012, followed by *post hoc* Bonferroni’s test, P = 0.0106; group x time interaction: F_(2,54)_ = 3.428, P = 0.0397) as well as between 15 and 21 dpi (RV-GFP: 109.13 ± 2.05%; AAV-Syn: 99.37 ± 0.51%, P = 0.0029) but not between 22 and 28 dpi (Fig. 6e). This suggests that the dynamic structural remodeling of new GCs takes place within the first three weeks of development, which is followed by structural stability with only minor refinement.

**Figure 7:**
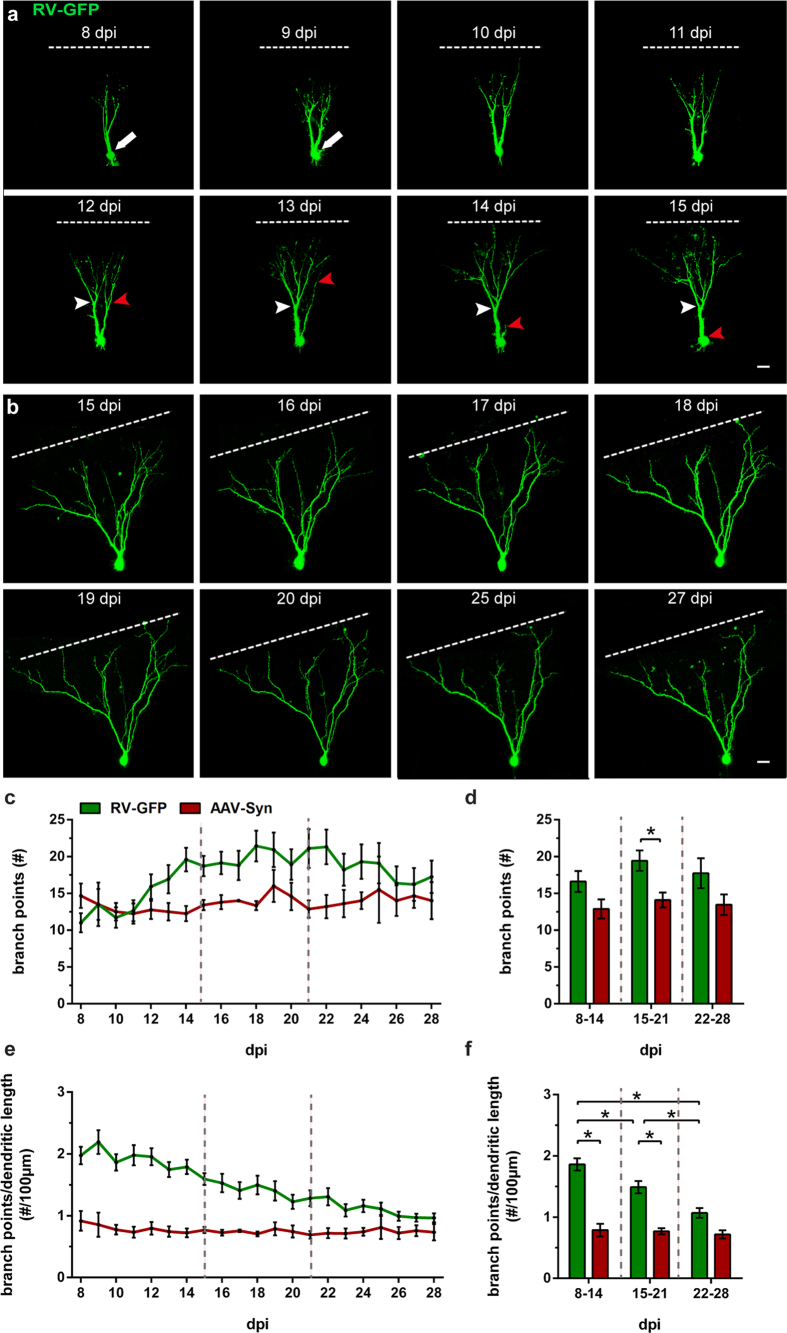
Newborn GCs undergo periods of branching and pruning while older GCs remain structurally stable. (**a**) A time-lapse image sequence of a newborn cell exemplifying the branching dynamics of the dendritic arbor during the second week of development. An example of the removal of a complete primary dendritic branch is displayed between 13 and 14 dpi (red arrowheads) while the other primary dendrite branched out and established a new dendritic structure (white arrowheads). Note the dislocation of the cell soma along the original primary dendrite toward the first branch point between 8–10 dpi (white arrow). (**b**) A time-lapse series that illustrates the dendritic growth phase which was pronounced until 20 dpi and was followed by a phase of stabilization (25–27 dpi). The growing dendrites reached the hippocampal fissure on 17 dpi (dotted line). (**c**) Daily mean values of the number of branch points. (**d**) At 15–21 dpi newborn GCs had significantly more branch points than older AAV-Syn GCs (two-way ANOVA with Bonferroni correction, P = 0.0470). (**e**) Daily mean values of the number of branch points per 100 μm of dendritic length. (**f**) The number of branch points per 100 μm TDL in newborn GCs was significantly reduced during each successive week, i.e. between the second and the third week (two-way ANOVA with Bonferroni correction, P = 0.0062), between the second and the fourth week (P < 0.0001) and between the third and the fourth week (P = 0.0061). Furthermore, newborn GCs had significantly more branch points per 100 μm TDL compared with older AAV-Syn GCs at 8–14 dpi (two-way ANOVA with Bonferroni correction, P < 0.0001) and at 15–21 dpi (P < 0.0001). (**d,f**) 8–14 dpi: n_RV-GFP_ = 17, n_AAV-Syn_ = 4; 15–21 dpi: n_RV-GFP_ = 18, n_AAV-Syn_ = 10; 22–28 dpi: n_RV-GFP_ = 11, n_AAV-Syn_ = 5. n represents number of cells (1–2 cells per culture). Error bars represent SEM. *P < 0.05. Scale bars: 10 μm.

The time-lapse series shown in Fig. 7a displays an example in which the dynamics of dendritic tree development are particularly intriguing. Between 8 and 9 dpi, we observed extensive branching and gradual translocation of the soma along the primary dendrite to the first branching point. The resulting two primary processes developed further branches and displayed a similar dynamic range as described above. However, in this particular case, we observed an entire primary dendritic branch while it disintegrated (Fig. 7a, red arrowheads). Between 12 and 13 dpi, one of the two primary dendrites lost several branches and appeared to become thinner (red arrowheads) while the other primary dendrite continued to expand and generate new branches (white arrowheads). By 14 dpi, the degenerating primary dendrite withdrew almost completely. The other branch, however, expanded further and displayed the dynamic process of extension and retraction of dendritic segments. On 15 dpi, the remaining primary dendrite had meanwhile developed even further and at this point represented the newly established shape and structure of the developing neuron.

Between 15 and 21 dpi, newborn GCs displayed continuing dynamics in dendritic growth and withdrawal of branches, as well as a further overall increase of dendritic tree size, although not as pronounced as during earlier time points (Fig. 7b). During this time, the cells contained the highest number of terminal segments overall. Between the third and the fourth week, newborn GCs exhibited an enhanced net loss of dendritic segments due to pronounced pruning. The phase of high dynamics in dendritic development was followed by a phase of stabilization and refinement. During the course of the fourth week (22–28 dpi), newborn cells displayed a stabilized dendritic tree in which established branches remained relatively stable and did not display additional branching or considerable growth. However, even during the fourth week when dendritic growth and retraction processes were less frequent, the cells did not become static but continued to exhibit small and localized adjustments to the dendritic arbor (Fig. 7b).

In regard to dendritic tree complexity, there was a continuous increase in branch points in newborn GCs during the second week of development, between 8 and 14 dpi, whereas in AAV-Syn-labeled GCs, the number of branch points stayed stable throughout the imaging time period (Fig. 7c–f). There were no significant differences in branch point number between the two cell populations in the second week. However, during the third week, between 15 and 21 dpi, newborn GCs displayed a significantly higher number of branch points (19.43 ± 1.35) compared with AAV-Syn-labeled GCs (14.09 ± 0.96, two-way ANOVA: group effect F_(1,59)_ = 7.963, P = 0.0065, followed by *post hoc* Bonferroni’s test, P = 0.0470). Interestingly, in the fourth week, newborn GCs showed a slight decrease in the amount of branch points and thus displayed no significant differences compared with older GCs at this time (Fig. 7d). When the number of branch points was normalized to each cell’s dendritic length (branch points per 100 μm dendritic length), we found a continuous decrease in newborn cells until the fourth week of development, i.e. between the second and the third week (8–14 dpi: 1.86 ± 0.10; 15–21 dpi: 1.49 ± 0.10; two-way ANOVA: time effect: F_(2,59)_ = 5.374, P = 0.0072, followed by *post hoc* Bonferroni’s test, P = 0.0062), between the second and the fourth week (8–14 dpi: 1.86 ± 0.10; 22–28 dpi: 1.07 ± 0.08; P < 0.0001) and between the third and the fourth week (15–21 dpi: 1.49 ± 0.10; 22–28 dpi: 1.07 ± 0.08; P = 0.0061; Fig. 7e,f). Moreover, newborn GCs displayed a significantly higher number of branch points per 100 μm dendritic length compared to older GCs during the second week (RV-GFP: 1.86 ± 0.10; AAV-Syn: 0.79 ± 0.09, two-way ANOVA: group effect F_(1,59)_ = 52.43, P < 0.0001, followed by *post hoc* Bonferroni’s test, P < 0.0001; group x time interaction: F_(2,54)_ = 3.759, P = 0.0291) and the third week (RV-GFP: 1.49 ± 0.10; AAV-Syn: 0.77 ± 0.05, P < 0.0001) but there were no significant differences between the two cohorts during the fourth week (Fig. 7f). This indicates that newborn cells underwent a process of over-branching, followed by dendritic pruning, until they reached a state of structural complexity comparable to older, mature GCs in the fourth week of development.

**Figure 8:**
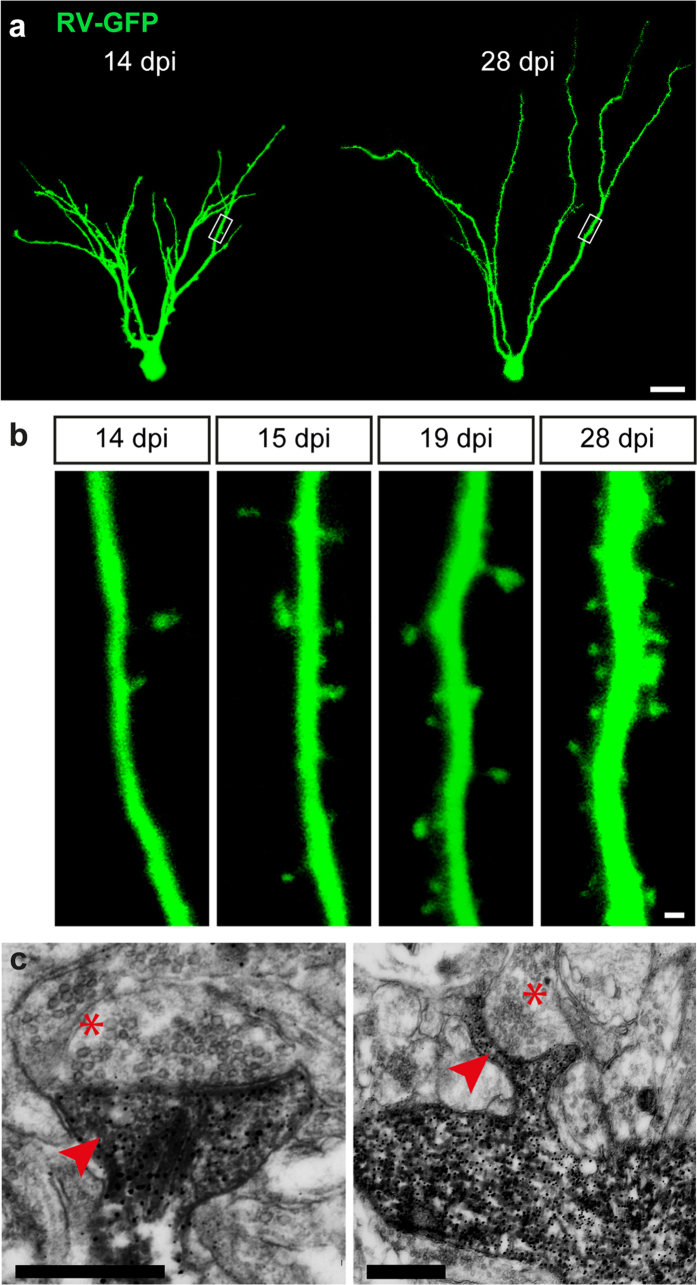
First spine-like structures appear between 14 and 16 dpi. (**a,b**) Time-lapse image series of a dendritic segment in the same area of a RV-GFP labeled cell at 14, 15, 19, and 28 dpi. The earliest presence of spine-like protrusions could be observed from 14 dpi on. Spine-like protrusions gradually increased in number and persisted throughout the remaining imaging period. (**c**) Electron micrographs depicting RV-GFP labeled dendritic segments (dark immunolabeling) at 19 dpi containing mushroom spines (arrowheads) that make synaptic contacts with GFP-negative axon terminals (asterisks) which exhibited numerous presynaptic vesicles. Scale bars: (**a**) 10 μm, (**b**) 1 μm, (**c**) 500 nm.

In time-lapse data between 13 and 24 dpi, we analyzed the timing of spinogenesis in newborn GCs (Fig. 8a,b). First occurrences of dendritic spine-like protrusions were detectable occasionally on 14 dpi, but more prominently on 15–16 dpi. In the following days, between 15 and 19 dpi, the number of spine-like structures increased considerably and by 19 dpi many of them exhibited hallmark characteristics of mature dendritic spines, such as a “neck” protruding from the dendrite and a prominent mushroom-shaped “head”^41^. From then on, spine numbers were further increasing continuously over time. By 24 dpi, all cells that were analyzed exhibited numerous spines throughout the dendritic arbor. In order to determine whether the newly formed spine-like protrusions established synaptic contacts with other cells, we performed electron microscopy of 19-day-old cultures (n = 3 cells from 2 animals) and found that, indeed, numerous RV-GFP-marked spines were involved in synapses with unlabeled axon terminals (Fig. 8c). These findings indicate that newborn GCs exhibit features of structural network integration in the slice culture system.

**Figure 9:**
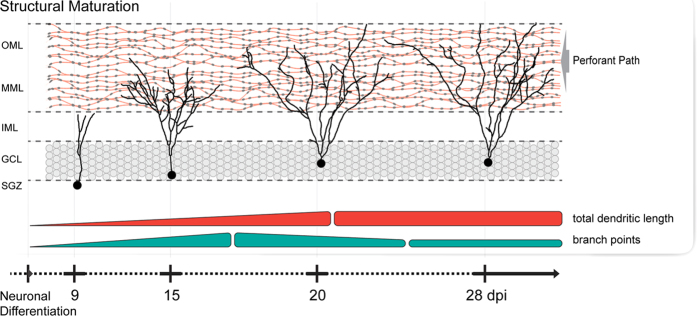
Time line of newborn GC development in OTCs. A schematic summary of our findings showing the temporal progression of newborn GC structural development in OTCs. During the second week of development, newborn cells exhibit a phase of dynamic dendritic extension and retraction, branching, and restructuring of the dendritic arbor with a high net increase in both total dendritic length and branch points. During the third week, newborn GCs display elaborate dendritic trees that reach a maximum level of complexity. In this time frame, the restructuring dynamics are less pronounced. Between the third and the fourth week, pronounced dendritic pruning is observed as the number of branches decreases. Finally, during the course of the fourth week, newborn GCs reach a phase of structural stabilization with only minor refinement to the dendritic arbor.

Taken together, our findings describe important hallmarks of structural development in individual newborn GCs over an extended period of time. The three weeks of observation correspond to three stages of dendritic maturation: A first phase of rapid, dynamic growth, followed by a second phase of high structural complexity and pruning, and a third phase of structural stabilization (Fig. 9; see also Supplementary Fig. S1).

## Discussion

In this study we used entorhino-hippocampal OTCs to investigate the maturation of postnatally born GCs in an organotypic environment. We implemented RV-labeling to identify postnatally born neurons as well as AAV-labeling of presumably prenatally born older GCs. Time-lapse imaging was performed to study the dynamics of dendritic maturation in the DG^42^. Using anterograde tracing, we showed that the dendrites of postnatally born GCs grow into an existing entorhinal termination zone, very similar to the situation in the adult rodent brain during adult neurogenesis. Postnatally born GCs showed an early period of fast growth, a phase of structural reorganization and pruning and, eventually, stabilization of their dendritic arbor. This sequence suggests that postnatally born GCs search for relevant inputs and optimize their connections in OTCs using a “trial and error” pathfinding method.

This study is one of the first to show that postnatal neurogenesis and the structural development of newborn GCs can be studied in OTCs and that it may be a useful and easily accessible tool to investigate mechanisms of GC integration over time within a standardized and controlled organotypic tissue culture system^29^. Previous work using a transgenic mouse model in which GFP expression in newborn neurons was induced by Cre recombinase during the first 3 DIV has demonstrated that morphological and physiological development of newborn GCs could be studied in OTCs over several weeks. These authors analyzed dendritic and axonal growth as well as electrophysiological properties of newborn GCs at 10 and 20 days and showed that new GCs matured structurally and functionally over time and were integrated into the hippocampal network^43^. In prior research into neurogenesis in OTCs involving treatment with BrdU or RV, labeling of newborn GCs was done after 2–2.5 weeks in culture, and analysis was performed up to 4 weeks following treatment^24^,^25^,^26^. It was shown that neurogenesis is found in OTCs shortly after explantation, and that newborn cells persist in the cultured environment. Using time-lapse imaging in OTCs from postnatal mice (P 3–5), Namba *et al*. showed primarily symmetrical division of neuron-committed cells from neural progenitors at DIV 2, thus revealing ongoing neurogenesis at least during the first days in culture^44^. However, with maturation of the cultures the generation of newborn cells subsides^45^,^46^, making it difficult to use older, i.e. more mature cultures to study adult neurogenesis at later time points. Although these earlier studies have reported on this limitation of the OTC system, we propose here that this system also has major advantages: OTCs prepared from early postnatal brains can be cultured for a period of more than 4 weeks, covering the time needed for the integration of newborn GCs. Furthermore, OTCs are excellent tools to study the integration of postnatally born neurons into an already existing dentate gyrus network as the entorhino-hippocampal connectivity is preserved^36^,^37^. This state is highly similar to the situation seen during adult neurogenesis, making it attractive to use this system to study the mechanisms of GC integration into the DG network.

In contrast to earlier studies using RV-labeling in OTCs, we have injected the RV construct at DIV 0 (P 4–5) into the cultures. At this time the so-called “tertiary dentate matrix” consists of a population of cells that proliferate and settle locally in the formative SGZ^14^. Thus, we RV-labeled neurons that are (i) postnatally born and (ii) eventually form the stem cell niche in the SGZ between P 7 and P 14^17^,^47^. Since the neurogenic niche of the SGZ, as it pertains to adult neurogenesis, is being established during this period^17^, and the cells in this population essentially undergo a similar developmental process as adult-born GCs^18^,^19^, investigation of GCs generated during the postnatal phase could serve as a good model to study mitosis, differentiation, structural maturation, and functional integration of newborn GCs into the DG neuronal network. Indeed, we verified and extended the earlier studies using DCX^48^, a well-established marker for immature neurons in postnatal and adult neurogenesis. In our preparations DCX was present in immature GCs of the SGZ, the inner parts of the GCL, as well as neurons located in the hilus at DIV 7. At DIV 28, DCX expression was limited to fewer cells that were located in the SGZ (Figs 1 and 2), thus revealing neurogenesis in postnatal OTCs. Previous studies have reported a substantial decrease in neurogenesis in OTCs during the first week of cultivation^45^,^46^. Since DCX expression can persist up to 3 weeks in individual GCs^21^,^22^,^48^,^49^, DCX-positive cells at DIV 28 may represent GCs that were generated at early time points but were still immature and continued to express DCX at the time of fixation. Alternatively, since neurogenesis does not cease completely in OTCs^24^,^25^,^45^,^46^, it could also be possible that a low number of GCs was generated at a later period. As in our cultures the number of DCX-expressing cells was much lower at the end of 4 weeks, our results reflect previous findings showing that the rate of neurogenesis in OTCs declines over time.

Prox1, a specific GC marker that is expressed early in precursor type 2b cells and persists throughout development and in mature GCs^32^,^50^,^51^,^52^, was prominent in the entire GCL between DIV 7–28 in postnatal OTCs. The majority of Prox1-positive cells exhibited co-localization with DCX or calbindin, a marker for mature neurons^53^ (Figs 1 and 2). Thus, we conclude that in our OTCs adult and newly born neurons exist side by side and that during a period of several weeks postnatal OTCs mature and achieve a more adult phenotype. However, by identifying prenatally born GCs (adult phenotype) and postnatally born GCs (immature phenotype), these two neuron types can be studied simultaneously and directly compared. Hence, postnatally generated GCs in the OTC system develop under conditions that are highly comparable to the situation during adult neurogenesis where adult GCs exist and newborn GCs grow into a neuropil filled by their dendrites and entorhinal axons.

Since we here make the case that postnatal GC development in OTCs can be used to study the integration of these cells into an established environment, it is important to consider the entorhinal axons, i.e. the presynaptic partners of the newborn GCs. As OTCs are explanted from the brain and thus at least some entorhinal fibers are transected^37^ in the process, we had to ensure the presence of these fibers in our culture preparations. Using anterograde tracing, we thus verified that the entorhinal fiber plexus is present in the OML of the DG. Earlier studies had reported that fibers from the EC are found within the molecular layer of the DG within 3 DIV^36^,^37^. We verified these reports in our cultures and compared the presence of fibers with the dendritic development of the RV-labeled GCs. Indeed, at our earliest investigation time point (DIV 8) entorhinal fibers were present in large numbers in the ML. The developing dendrites of the RV-labeled cells, however, had not yet entered this zone, demonstrating that their elongation will have to occur into the already present entorhinal fiber plexus. This was indeed the case as we verified by tracing older cultures (DIV 20). We conclude from these data that postnatally born GCs grow into an existing entorhinal termination field.

The structural development of RV-labeled GCs has been previously described in fixed tissue from intact animals^18^,^19^,^23^,^33^. These authors reported newborn GCs with short processes and round somata around 7 dpi^18^. By 14 dpi RV-labeled GCs exhibited a dendritic tree that reached the middle molecular layer (MML) of the DG^18^,^19^. Starting at 21 dpi, adult-born GCs had features of mature GCs^18^,^19^,^23^,^33^ and essentially no change was seen thereafter^19^,^23^. Since these data are from animals sacrificed at different time points post RV injection, the dynamics of these changes were not analyzed in these studies. Using the OTC preparations and time-lapse imaging we found that postnatally born GCs undergo three maturation stages that differ with regard to their dynamics. During the first phase, which corresponds to the second week post injection, postnatally born GCs showed the highest dynamic structural changes. Notably, although the dendritic tree was growing in overall dendritic length and branching out toward the ML, dendritic deconstruction was also observed: Already established segments, and in some cases large parts of the arbor arising from proximal (i.e. lower order) dendrites, were withdrawn (see Figs 5, 6 and 7). During the second phase, which corresponds to the third week post injection, postnatally born GCs exhibited a sizable arbor with a considerable degree of complexity. Moreover, the dendrites started to exhibit spine-like structures, usually at 14–16 dpi (Fig. 8b). These data are in line with previous findings that described newborn GCs in adult mice to contain spines at 16 dpi^19^. In ultrastructural studies, Toni, *et al*. (2007) described mature synaptic input on RV-labeled adult-born mouse GCs at 30 dpi *in vivo*, characterized by the presence of a postsynaptic density (PSD), at least four presynaptic vesicles within 100 nm of the presynaptic membrane, and a distinct synaptic cleft^54^. Along those lines, we performed ultrastructural imaging on 19 dpi and observed that RV-GFP-labeled spines made synaptic contacts with axon terminals from unlabeled cells (Fig. 8c). Even as spines and synaptic contacts were established during this time period, the dendritic tree was still shaped and pruned although these adaptive processes were less frequent and less extensive than during phase 1. Finally, during the third phase, which corresponds to the fourth week and later, GC dendrites were stable with only limited, i.e. local, dendritic reorganization occurring. The overall morphology and dynamics of these 3–4-week-old RV-labeled cells in our rat OTCs was found to be well comparable to cells in the adult mouse DG *in vivo*^55^. Thus, we here report a highly dynamic integration of GCs into their termination zone which is characterized by both, dendritic growth as well as dendritic retraction. The integration of GC dendrites into the hippocampal network hence shows considerable similarities to a complex pathfinding process rather than a simple growth process. This mechanism may be highly efficient to guide dendrites to their correct locations and, possibly, their correct synaptic partners. Since GC dendrites require entorhinal input to be maintained^56^, we speculate that only those dendritic segments that attract entorhinal input will be preserved, whereas segments that fail to do so will be pruned. Thus, new GCs might find their way using a “trial and error” mechanism.

Our data are the first to describe the dynamic integration of postnatally born GC dendrites into the entorhinal termination zone in OTCs in detail and over an extended time period. In an earlier study, time-lapse imaging of RV-labeled cells was performed in slice cultures prepared from adult mice^28^. Because of the limitations of cultures prepared from adult animals, however, these authors could only image the first 9 DIV. In their preparations, an apically extending dendrite was observed, but the long-term fate of this dendrite could not be ascertained. In recently published work by Gonçalves and colleagues (2016), the dendritic dynamics of GCs born in the adult mouse brain were described using 2-photon imaging^55^. These authors showed growth and pruning of adult born GC dendrites *in vivo*, similar to what we report here for the OTC preparation. We conclude from this comparison with the recent *in vivo* data that the integration of postnatally born GCs into the DG-entorhinal network *in vitro* exhibits a similar dynamic pattern as *in vivo*. Thus, the OTC preparation may be a highly convenient and effective way to simulate the integration of new neurons in an existing neural network *in vitro* and to use it as an assay system to generate hypotheses that can later be tested under *in vivo* conditions.

## Materials and Methods

### Organotypic entorhino-hippocampal slice culture preparation

Animal care and experimental procedures were performed in agreement with the German law on the use of laboratory animals (animal welfare act; TierSchG; §4 par 3) and approved by the animal welfare officer of Goethe-University, Faculty of medicine. Organotypic entorhino-hippocampal slice cultures (OTCs; 300 μm thickness) were prepared from 4–5 day old (P 4–5) Sprague Dawley rats of either sex using the interface method^30^. Briefly, brains were sliced horizontally into 300 μm thick sections using a vibratome (Leica VT1000S) at low speed (0.10–0.15 mm/s) and high vibration frequency (80–90 Hz). The hippocampi with attached entorhinal cortices were dissected with scalpels and transferred onto sterile membrane culture inserts (Millicell-CM, Millipore; 0.4 μm pore size, 30 mm diameter). These were placed into pre-incubated six-well plates containing slice culture incubation medium (1 ml per well) that consisted of 42% MEM, 25% Basal Eagle Medium containing Earle’s salts, 25% heat-inactivated normal horse serum, 0.65% glucose, 25 mM HEPES, 0.1 mg/ml streptomycin, 100 U/ml penicillin, 0.15% sodium bicarbonate, and 2 mM glutamax, adjusted to pH 7.30. Cultures were kept *in vitro* in a humidified incubator (95% air, 5% CO_2_, at 35 °C), and the medium was changed every 2 to 3 days until further processing.

### Viral transduction and retroviral labeling

To label newly born cells in OTCs that would allow visibility of entire cells, cultures were transduced with a retroviral vector based on the Moloney murine leukemia virus (MMLV) containing the gene for green fluourescent protein (GFP) under the CAG promoter^19^,^33^. The retrovirus (RV) infected only dividing cells because the pre-integration complex that contains the viral DNA cannot permeate the intact nuclear membrane of a cell^57^,^58^. Local injections were performed using a NanoFil syringe (World Precision Instruments). Approximately 0.2 μl of virus solution (10^5^ colony forming units; CFU) was injected directly into each culture (into or near the DG) using a 35-gauge beveled needle (World Precision Instruments). The structure of OTCs was visualized with a stereo microscope (Zeiss Stemi 2000-C) that was equipped with a camera (ColorView II soft imaging system) and the software AnalySIS 2677 (Olympus) which allowed precise injection of the RV solution into the area of interest. The incubation medium was changed every 2 days after transduction until OTCs were fixed.

In a subset of OTCs, a cocktail of the RV-CAG-GFP and an adeno-associated viral (AAV) vector encoding for tdTomato under the synapsin promoter was injected in the same manner. This was done in order to concurrently label newborn cells as well as older, more mature cells that served as a control group.

### Time-lapse imaging of slice cultures

Live imaging of slice cultures was performed as previously described^59^. The membrane insert with the cultures was placed into a 30 mm petri dish that contained warm (37 °C) imaging medium which consisted of NaCl 129 mM, KCl 4 mM, MgCl2 1 mM, CaCl2 2 mM, glucose 4.2 mM, HEPES 10 mM, Trolox 0.1 mM, streptomycin 0.1 mg/ml, penicillin 100 U/ml; pH 7.4. The osmolarity of the imaging medium was adjusted with sucrose to the osmolarity of the incubation medium. Imaging was done with an upright confocal microscope (Zeiss, Pascal; 488 nm excitation laser) equipped with a temperature-regulated stage (37 °C), using a 10x water immersion objective lens (0.3NA; Zeiss) to visualize slice cultures and to identify RV- or AAV-labeled dentate granule cells within the slice. Image stacks (30–40 images per stack; z-axis interval between consecutive frames: 2 μm) of individual GCs were obtained with a 40x water immersion objective lens (0.8NA; Zeiss) at a resolution of 1024 × 1024 pixels. Dendritic segments and spines were imaged with a 63x water immersion objective lens (0.9NA; Zeiss) with a 4x field zoom (15–25 images per stack; z-axis interval between consecutive frames: 0.5 μm). OTCs were imaged once per day for less than 10 minutes per culture in order to keep exposure time and phototoxic damage minimal.

Images were edited with Fiji (Image Processing and Analysis in Java, version 1.48)^60^ and/or Adobe Photoshop CS6 version 13.0 × 64 for contrast, background reduction, rotation, and selection of region of interest. Figures were prepared with Adobe Illustrator CS6 version 16.0.0.

### 3-D reconstruction of labeled GCs and morphological analysis

Complete dendritic trees of GFP- and tdTomato-labeled GCs were reconstructed from confocal image stacks (30–40 images per stack; z-axis interval between consecutive frames: 2 μm, magnification: 40x, see above) with the software TREES Toolbox^61^,^62^ in Matlab (MathWorks, Natick, MA, U.S.A.). The cells’ dendrites were traced by placing anchor nodes from the soma to their termination points in three dimensions. Upon completion, reconstructed trees were resampled to an internode distance of 1 μm. Dendritic diameter (“quaddiameter tree”) and soma thickness (“soma tree”) were adjusted according to the image. Morphological analyses were performed using Matlab (MathWorks, Natick, MA, U.S.A.), the TREES Toolbox^62^, and custom written algorithms.

### Immunocytochemistry

Cellular phenotypes were determined using several chemical cell type markers, including the immature neuronal marker doublecortin (DCX), calbindin (CB), which labels mature neurons, as well as the GC marker Prospero-related homeobox 1 gene (Prox1)^50^,^53^,^63^. OTCs, still attached to the membrane inserts, were fixed in 4% paraformaldehyde/4% sucrose in PBS (pH 7.40) for 1 hour at room temperature and 2% PFA/30% sucrose in PBS overnight at 4 °C. Following a wash in TBS (0.1 M TRIS in dH_2_O, pH 7.40) + 0.01% NaN_3_, cultures were sliced into 50 μm thick sections using a vibratome (Leica VT1000S) at a speed of 0.25–0.50 mm/s and a frequency of 80 Hz. The free-floating sections were washed three times in TBS + 0.01% NaN_3_ for 5 minutes and blocked with 5% bovine serum albumin (BSA) + 0.5% Triton X-100 in TBS for 1 hour at room temperature. Subsequently, sections were incubated with appropriate primary antibodies in TBS + 0.01% NaN_3,_ 0.1% Triton X-100, and 1% BSA overnight at room temperature. The following primary antibodies were used against: DCX (goat, polyclonal, 1:500, Santa Cruz), calbindin (mouse, monoclonal, 1:1000, Swant), Prox1 (rabbit, polyclonal, 1:1000, ReliaTech), and green fluorescent protein (GFP, mouse, polyclonal, 1:1000, Swant). Sections were washed in TBS + 0.01% NaN_3_ three times for 5 minutes before they were treated with fluorescent dye conjugated secondary antibodies (1:1000; Alexa 488, 568, and 633, Vector Labs) for 4 hours at room temperature. Finally, sections were mounted with DAKO fluorescent mounting medium (Dako Cytomation). Results were analyzed by confocal microscopy (Nikon Eclipse 80i).

### Anterograde axonal tracing

To label entorhinal projection fibers, a few crystals of the neurotracer Mini Ruby, a biotin-conjugated 10 kD dextran amine (MoBiTec, Göttingen, Germany), were placed onto layers II and III of the entorhinal cortices in RV-treated and untreated OTCs on DIV 3 and DIV 15. Cultures were fixed on DIV 8, DIV 17, or DIV 20 and processed for immunocytochemistry as described previously.

### Confocal microscopy and analysis

High resolution (1024 × 1024 pixel) confocal images of fixed histological sections were obtained with a confocal laser scanning microscope (Nikon Eclipse 80i) equipped with a camera (Nikon D-Eclipse C1), using the software EZ-C1 3.60. Overlapping image stacks (4–5 stacks per OTC section; 10–20 images per stack; z-axis interval between consecutive frames: 1 μm) were recorded using a 10x, 20x, or 40x oil immersion lens (Nikon, numeric aperture 1.3). Image stacks were analyzed with the Fiji software. Images were edited with Fiji and/or Adobe Photoshop CS6 version 13.0 × 64 for contrast, rotation, and selection of region of interest. Figures were prepared with Adobe Illustrator CS6 version 16.0.0.

### Electron Microscopy

Slice cultures were fixed for 1.5 h in 0.1 M sodium cacodylate buffer (CB) containing 4% paraformaldehyde and 2% glutaraldehyde. Fixed cultures were resliced to 50 μm with a vibratome (Leica VT1000S) at a speed of 0.25–0.50 mm/s and a frequency of 80 Hz. Following a TBS wash, free floating sections were blocked with 5% BSA in 0.1% NaBH4 (Sigma-Aldrich) for 1 h at room temperature. For detection of GFP-labeled cells, sections were first incubated with anti-GFP (goat, 1:500; Acris, Herford, Germany) primary antibody in 2% BSA in 0.1 M TBS for 18 h at room temperature, followed by an incubation with a biotinylated anti-goat IgG (1:200; Vector Laboratories, Burlingame, CA) secondary antibody for 60 min at room temperature. After washing in TBS, sections were incubated in avidin-biotin-peroxidase complex (ABC-Elite, Vector Laboratories) for 90 min at room temperature and reacted with a diaminobenzidine solution (Vector Laboratories) for 2–15 min at room temperature. Sections were silver-intensified by incubation in 3% hexamethylenetramine (Sigma-Aldrich), 5% silvernitrate (AppliChem) and 2.5% di-sodiumtetraborate (Sigma-Aldrich) for 10 min (60 °C); 0.05% tetrachlorogold (AppliChem) solution for 3 min, and 2.5% sodium thiosulfate (Sigma-Aldrich) for 3 min. Following each step, sections were washed in distilled water.

After staining, sections were washed in 0.1 M CB, osmicated for 30 min with 0.5% OsO4 (Plano, Wetzlar, Germany) in 0.1 M CB, dehydrated for 60 min with 1% uranyl acetate (Serva, Heidelberg, Germany) and 70% ethanol in H2O, and embedded in Durcupan (Sigma-Aldrich) for ultrathin sectioning (60 nm) using Ultracut (LeicaUCT). Sections were collected on singleslot Formvar-coated copper grids that were contrast enhanced with lead citrate for 4 min and examined using a Zeiss electron microscope (Zeiss EM 900) at 20,000× magnification.

### Statistical analysis

Statistical analysis and data visualization were done with Microsoft Excel (Microsoft, Redmond, Washington, USA) and GraphPad Prism 6 (Graphpad Software, San Diego, CA, USA). Statistical testing was performed with the two-way ANOVA followed by a *post hoc* Bonferroni test. Significance level was set to P < 0.05, denoted by an asterisk (*). Results are expressed as mean ± SEM.

## Additional Information

**How to cite this article**: Radic, T. *et al*. Time-lapse imaging reveals highly dynamic structural maturation of postnatally born dentate granule cells in organotypic entorhino-hippocampal slice cultures. *Sci. Rep.* **7**, 43724; doi: 10.1038/srep43724 (2017).

**Publisher's note:** Springer Nature remains neutral with regard to jurisdictional claims in published maps and institutional affiliations.

## Supplementary Material

Supplementary Information
