# Supplementary material for: Time-lapse imaging reveals highly dynamic structural maturation of postnatally born dentate granule cells in organotypic entorhino-hippocampal slice cultures
Source: Sci Rep. 2017 Mar 3;7:43724. doi: 10.1038/srep43724 (PMC5335612; doi:10.1038/srep43724)
Supplement: Supplementary Information [file srep43724-s1.pdf]

Time-lapse imaging reveals highly dynamic structural maturation of postnatally born dentate granule cells in organotypic entorhino-hippocampal slice cultures

Tijana Radic[1*], Tassilo Jungenitz[1], Mathias Singer[1], Marcel Beining[1,2,3], Hermann Cuntz[2,3], Andreas Vlachos[1§], Thomas Deller[1] and Stephan W. Schwarzacher[1]

[1] Institute of Clinical Neuroanatomy, NeuroScience Center, Goethe University, Frankfurt am Main, Germany

[2] Ernst Strüngmann Institute (ESI) for Neuroscience in Cooperation with Max Planck Society, Frankfurt am Main, Germany

[3] Frankfurt Institute for Advanced Studies (FIAS), Frankfurt am Main, Germany

[§] Current address: Institute for Anatomy II, Faculty of Medicine, Heinrich-Heine-University, Düsseldorf, Germany

* Corresponding author: Tijana Radic, Institute of Clinical Neuroanatomy, NeuroScience Center, Goethe University, Theodor-Stern-Kai 7, D-60590 Frankfurt am Main, Phone: +49 (0)69 6301 6914, Fax: +49 (0)69 6301 6425, E-mail: tijica@gmail.com

Supplementary Fig. S1. Time-lapse imaging of RV-labeled postnatally born GCs. Successful RV-labeling of postnatally born GCs in OTCs enabled live imaging of individual cells over extended periods of time. Daily time-lapse imaging sequences were performed between 8-28 dpi, up to 20 consecutive days. Three-dimensional computer reconstructions enabled detailed analysis of morphological development of individual newborn GCs. Based on immunostainings of fixed OTCs on 28 dpi, the location of the hippocampal fissure could be determined *post hoc* (dotted line). Scale bar: 10 μm.

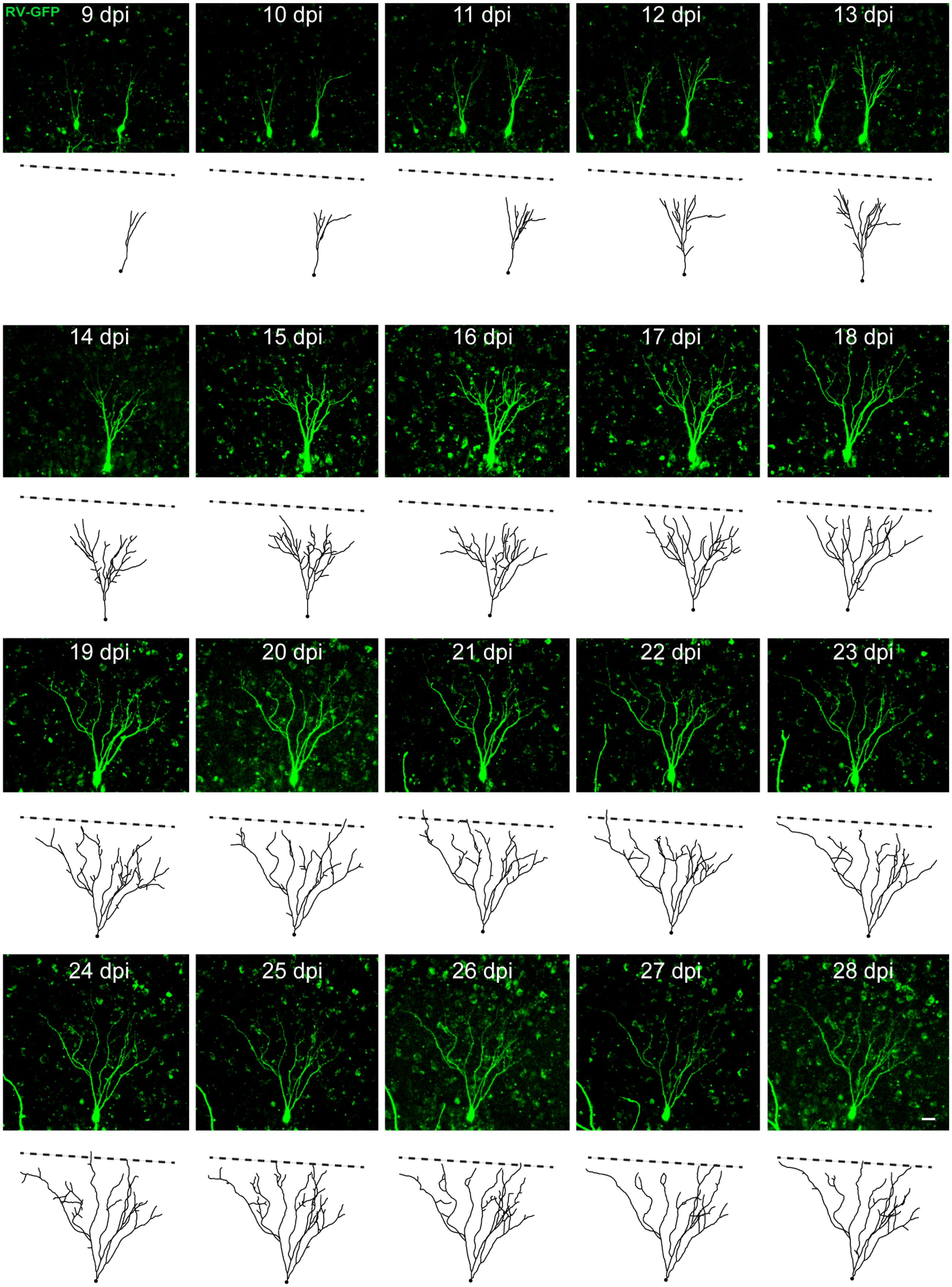
RV-GFP
9 dpi
10 dpi
11 dpi
12 dpi
13 dpi
14 dpi
15 dpi
16 dpi
17 dpi
18 dpi
19 dpi
20 dpi
21 dpi
22 dpi
23 dpi
24 dpi
25 dpi
26 dpi
27 dpi
28 dpi
